# Boys Equal Girls

**DOI:** 10.1111/cen.15234

**Published:** 2025-03-16

**Authors:** Tim Cheetham

**Affiliations:** ^1^ Great North Children's Hospital and Newcastle University Newcastle upon Tyne UK

What is normal? Reference standards are a core component of endocrine practice but they come with caveats. Lying below the 2nd or above the 98th centiles—or lying below or above two standard deviations outwith the mean—does not automatically mean that there is underlying pathology. The ‘lines in the sand’ associated with biochemical reference ranges can be helpful but clinicians will be aware that normal ranges do not encompass all healthy individuals.

Many physiological processes are subject to a similar yes/no classification with the paediatrician deciding whether a child is in puberty or not on the basis of the clinical assessment. The onset of puberty reflects rising gonadotropin secretion with ovarian oestrogen production and subsequent breast development being the key clinical marker of pubertal onset in girls and testicular enlargement > 3mLs the key marker in boys. Breast development on average occurs before the attainment of the 4 mL ‘pubertal’ testicular volume (just over 11 years of age vs. 12 years) with the implication being that puberty begins earlier in girls than boys [1, 2, 3].

This matters from a clinical perspective because the earlier that puberty begins in advance of normal timing, the more likely it is that there is an underlying pathological explanation for this. Irrespective of the underlying mechanism, there are effective treatments, notably GnRH analogues, that can safely put puberty on hold.

Pubertal assessment of girls can be complicated by an increased body mass index and a degree of associated lipomastia and there are complicating factors in boys as well. The prepubertal testicle will vary in size between healthy individuals—a normal continuum. Hence a testicular volume of 2.5 mLs in one individual can presumably equate to the same physiological stage of development as a 1.5 mL volume in someone else. Perhaps a 3.5 mL volume (pubertal) in one child will be equivalent from a developmental perspective to a 2.5 mL volume (prepubertal) in someone else. Then there are factors such as the means of assessment—for example clinical examination or ultrasonography—that need to be taken into account. It is not uncommon for my clinical assessment to be different to that of my colleagues, an observation reinforced by more objective data collection [4].

An assessment of pubertal stage of development can be bolstered by blood tests such as baseline gonadotropins, their response to LHRH stimulation and by measuring sex steroid concentrations but the results of these investigations also form a continuum rather than neatly separating pre‐pubertal from pubertal.

In this issue of Clinical Endocrinology Demir and colleagues from Finland [5] have investigated urinary luteinizing hormone (U‐LH) gonadotropin production in a large cohort of boys and girls. The authors conclude that measuring U‐LH concentrations is a useful approach to establishing whether a child is in puberty or not. Day to day variation in the first morning urine samples was substantial but did not prevent the investigators from categorising the majority of patients correctly into prepubertal verses ‘highly likely’ pubertal/pubertal. Interestingly, U‐LH concentrations indicated that participants were in puberty 1–2 years in advance of the appearance of clinical signs. The authors conclude that U‐LH can be used as a screening test to classify children into prepubertal, peri‐pubertal and pubertal groups and hence the measurement of U‐LH could potentially help to prevent more expensive, time consuming and potentially uncomfortable investigations. Nevertheless, when faced with a child in clinic with potential early or precocious puberty paediatricians may still prefer blood samples so that other markers of pubertal development can be assessed as well. The logistical issues associated with obtaining a pertinent urine sample at the time of the clinic appointment introduce an additional layer of complexity and it sounds as though the assay used by the investigators is now obsolete. Whether U‐LH proves to be anything more than a research tool in the longer term remains to be seen.

An interesting component of the paper by Demir and colleagues is the fact that it underlines how the onset of increased gonadotropin production at puberty actually occurs at a similar time in boys as girls. In this cohort the increase in mean total U‐LH concentrations appeared shortly after the age of 10 years in males and females and so the sexes have more in common than was previously thought to be the case. Whilst the attainment of a 4 mL testicular volume in boys may occur later than the presence of clinical breast tissue in girls, the physiological process underpinning these changes will have started many months previously when the ‘4 mL’ testicle was much smaller. Demir and colleagues show us that there will already be an incremental ‘pubertal’ background rise in LH production in some boys with a 1 mL testicular volume who from a more traditional clinical perspective are not yet in puberty. This study has helpfully educated biologists and health professionals about the events taking place before the > 3 mL testicular ‘line in the sand’.
